# Hematopoietic Gene Expression Regulation Through m^6^A Methylation Predicts Prognosis in Stage III Colorectal Cancer

**DOI:** 10.3389/fonc.2020.572708

**Published:** 2020-09-30

**Authors:** Zheng Zhou, Shaobo Mo, Ruiqi Gu, Weixing Dai, Xinhui Zou, Lingyu Han, Long Zhang, Renjie Wang, Guoxiang Cai

**Affiliations:** ^1^Department of Colorectal Surgery, Fudan University Shanghai Cancer Center, Shanghai, China; ^2^Department of Oncology, Shanghai Medical College, Fudan University, Shanghai, China; ^3^School of Public Health, Shanghai Medical College, Fudan University, Shanghai, China; ^4^Department of Cancer Institute, Fudan University Shanghai Cancer Center, Fudan University, Shanghai, China

**Keywords:** colorectal cancer, stage III, m^6^A, signature, prognosis

## Abstract

**Background:**

Methylation of N6 adenosine (m^6^A) plays important regulatory roles in diverse biological processes. The purpose of this research was to explore the potential mechanism of m^6^A modification level on the clinical outcome of stage III colorectal cancer (CRC).

**Methods:**

Gene set variation analysis (GSVA) and gene set enrichment analysis (GSEA) were adopted to reveal the signal pathway which was most likely affected by m^6^A methylation. The linear models for microarray data (LIMMA) method and the least absolute shrink-age and selection operator (LASSO) Cox regression model were used to identify the signature. The signature can sensitively separate the patients into high and low risk indicating the relapse-free survival (RFS) time based on time-dependent receiver operating characteristic (ROC) analysis. Then, the multi-gene signature was validated in GSE14333 and the Cancer Genome Atlas (TCGA) cohort. The number of the samples in GSE14333 and TCGA cohort are 63 and 150. Finally, two nomograms were set up and validated to predict prognosis of patients with stage III CRC.

**Results:**

The hematopoietic cell lineage (HCL) signaling pathway was disclosed through GSEA and GSVA. Seven HCL-related genes were determined in the LASSO model to construct signature, with AUC 0.663, 0.708, and 0.703 at 1-, 3-, and 5-year RFS, respectively. Independent datasets analysis and stratification analysis indicated that the HCL-related signature was reliable in distinguishing high- and low-risk stage III CRC patients. Two nomograms incorporating the signature and pathological N stage were set up, which yielded good discrimination and calibration in the predictions of prognosis for stage III CRC patients.

**Conclusions:**

A novel HCL-related signature was developed as a predictive model for survival rate of stage III CRC patients. Nomograms based on the signature were advantageous to facilitate personalized counseling and treatment in stage III CRC.

## Background

In 2019, the nation’s 14.8 million new colorectal cancer (CRC) cases made it the most common cancer of digestive tract, with 146 deaths per day ranking third among all malignant tumors in the United States (1). Closely related to economic developments, CRC has emerged as a critical public health problem in China as the living standard of its people improves, and the incidence of CRC was about 37.6/100,000 in 2016, ranking third likewise (2). More than 50% of patients with CRC are diagnosed at or beyond stage III. Therefore, distant metastasis occurred and their 5-year survival rate drops to 10%. Adjuvant chemotherapy (ACT) combined with surgery is the prominent treatment to enhance survival for stage III CRC patients (3). Many variables contribute to the prognosis of stage III CRC patients. For instance, the number of negative lymph nodes is a significant prognostic factor for patients with stage III CRC (4). Perineural invasion (PNI) is also a prognostic factor. Stage III CRC patients with PNI are more likely to have metastasis and recrudesce (5).

According to current NCCN guidelines, FOLFOX (fluorouracil, leucovorin, and oxaliplatin) or CAPOX (capecitabine and oxaliplatin) has become the first-line ACT for stage III CRC patients. It has been proved that stage III CRC patients with proper ACT have a survival advantage compared to those without ACT (6). Based on the results carried out by the International Duration Evaluation of Adjuvant Therapy (IDEA) collaboration, for low-risk (T1-3, N1) stage III CRC patients, the optimal ACT options are 3 months of CAPOX or 3 to 6 months of FOLFOX. 6 months of FOLFOX or 3 to 6 months of CAPOX is suitable for the high risk (T4, N1-2 or T any, N2) stage III patients (7). However, there is a lack of effective molecular markers for the prognosis of stage III CRC.

N6-methyladenosine (m^6^A) is one form of RNA modifications. M^6^A RNA methylation, which are widely found in the various types of RNA, is recognized as the most prominent and abundant form of internal modifications in eukaryotic cells. M^6^A modification is regulated by methyltransferases, demethylases and binding proteins, which can be also called “writers,” “erasers” and “readers.” It has been reported that the m^6^A regulators play a crucial role in a variety of biological functions in post-transcriptional regulation of gene expression (8). Increasing evidence demonstrated that dysregulated expression and genetic changes of m^6^A regulators were correlated with the disorders of multiple biological process including abnormal cell death and proliferation, developmental defects, tumor malignant progression, and immunomodulatory abnormality. Previously, researchers unraveled the correlation between the genetic alterations of m^6^A regulatory genes and TP53 pathway in the processing of acute myeloid leukemia (9). Recently, research into the gastric carcinoma proved that m^6^A modification in cancer tissue had a close relationship with tumor microenvironment (10). Certain single-nucleotide polymorphisms (SNPs) in m^6^A modification genes were also proved to have correlation with the formation of CRC (11). To conclude, m^6^A modifications not only correlated with the hematologic tumor, but it might also provide novel insight into the classification and precise treatment toward gastrointestinal carcinoma.

Hematopoietic stem cells (HSCs) are self-renewing and have the potential to become different progenitor cells. The differentiation mainly follows two pathways, which are lymphoid and myeloid pathways. In the lymphoid pathway, the common lymphoid progenitor cells differentiate into immune cells and in myeloid pathway, the progenitor cells differentiate into granulocytes, monocytes, erythrocytes and platelets. Hematopoietic cell lineage (HCL) pathway has both intercellular and extracellular factors via transcription as well as post transcription level. DNA methylation, histone modifications, small non-coding RNAs are involved in post transactional regulation (12). However, the correlation between HCL pathway and CRC still needs further investigation.

In this research, CRC patients’ gene expression microarray data and clinicopathological information were adopted from the Gene Expression Omnibus (GEO) for identifying different m^6^A modification patterns mediated by m^6^A regulators (13). Using principal component analysis (PCA), Gene Set Variation Analysis (GSVA) and Gene Set Enrichment Analysis (GSEA), a seven-HCL related regulators was identified from the GSE39582 and GSE14333, downloaded from GEO. The GSVA is a non-parametric unsupervised method for assessing gene set enrichment (GSE) in gene expression microarray and RNA-seq data. In contrast to most GSE methods, GSVA performs a change in coordinate systems, transforming the data from a gene by sample matrix to a gene set by sample matrix. Thereby allowing for the evaluation of pathway enrichment for each sample. This transformation is done without the use of a phenotype, thus facilitating very powerful and open-ended analyses in a now pathway centric manner (14). Then, we constructed a predictive gene signature and verified the results in GSE14333 and the Cancer Genome Atlas (TCGA) cohorts. Eventually, nomograms based on the prognostic signature and clinicopathological characteristics was constructed to assess prognosis in stage III CRC patients.

## Materials and Methods

### Data Selection

A total of 21 m^6^A regulators were extracted from two independent GSE datasets, GSE39582 and GSE14333, downloaded from the GEO database^1^, for identifying different m^6^A modification patterns mediated by 21 m^6^A regulators. These 21 m^6^A regulators included 8 writers (*METTL3, METTL14, RBM15, RBM15B, WTAP, KIAA1429, CBLL1, ZC3H13*), 2 erasers (*ALKBH5, FTO*) and 11 readers (*YTHDC1, YTHDC2, YTHDF1, YTHDF2, YTHDF3, IGF2BP1, HNRNPA2B1, HNRNPC, FMR1, LRPPRC, ELAVL1*) (Supplementary Table 1) (10). The 87 genes in Supplementary Table 2 were derived from “KEGG_ HEMATOPOIETIC_CELL_LINEAGE” gene list within 186 Kyoto encyclopedia of genes and genomes (KEGG) gene sets of canonical pathways, download from the MSigDB of GSEA databas^2^. Expression data and clinical information were downloaded from GEO database and robust multichip average method was applied in normalizing the raw microarray data (13). UCSC Xena^3^were the source of the TCGA clinical information and genome data. GSE39582 is the largest, most comprehensive and most complete data series in the GEO dataset. It contains 23495 genes’ expression information of 585 patients. The GSE14333 data series contains genes expression information of 290 patients sequenced by same measuring method as GSE39582. In this research, we only extracted the stage III CRC patients’ data, which are 205 and 91, respectively. The detailed and demographical information is listed in the Supplementary Table 3.

### PCA, GSVA, and GSEA

**FIGURE 1:**
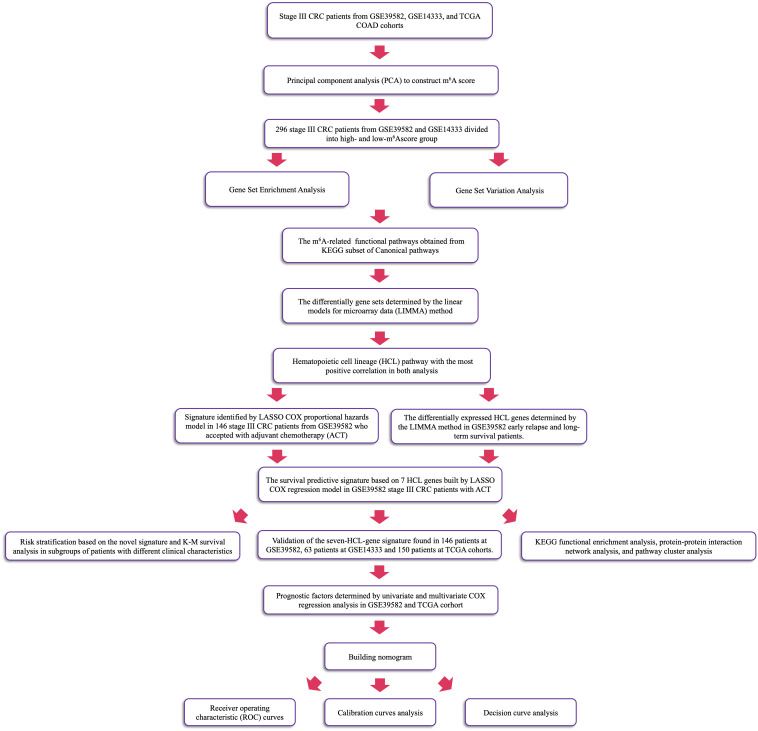
The workflow of identification of CRC ACT-related seven-HCL signature.

To quantify the m^6^A modification patterns of individual tumor, the m^6^Ascore, a set of scoring system was constructed to evaluate the m^6^A modification pattern of individual patients with CRC. This research performed PCA on the expression levels of 21 m^6^A regulators, which were identified as principal components, in GSE39582 and GSE14333 to reduce the number of dimensions and construct m^6^Ascore. This method had advantage of focusing the score on the set with the largest block of well-correlated (or anticorrelated) genes in the set, while down-weighting contributions from genes that do not track with other set members. The median of sums of 21 principal components in 296 samples was calculated as the cut-off points to divide patients into two m^6^A clusters. Using the KEGG gene sets as the reference gene set and setting the *p* value < 0.05, we conducted GSVA to measure the signaling pathway variation score for each sample in stage III CRC by using “GSVA” R package (14). In this research, enrichment score was calculated as the magnitude difference between the largest positive and negative random walk deviations. GSEA was also performed to analyze difference between CRC patients’ m^6^A subgroups via “javaGSEA” to obtain GSEA result with the same data sets (15). Then, linear models for microarray data (LIMMA) method was used to sort out the pathway with the most positive correlation. The detailed workflow is shown in Figure 1.

### Construction of the Predictive Gene Signature

Patients suffering from early recurrence within 1 year after primary resection was classified as early relapsing group. Based on the “glmnet” package in R, we searched optimum predictive genes for GSE39582 CRC samples by applying pathway brought from the results of GSVA and GSEA (16), and using least absolute shrink-age and selection operator (LASSO) Cox regression analysis. In LASSO regression, recurrence-free survival, which was also used to determine the ACT response, was identified as patients’ outcomes. Besides, LIMMA method was introduced to conduct an analysis of differentially expressed genes (DEGs) between early recurrence and long-term survival patients (no relapse after at least 5 years after the surgery) (17), with *p* < 0.05 and fold change ≥1.1. The number of patients in the early relapsing group in the GSE39582 was 26 while the number of patients in the long-term survival group was 56. Considering the results of LASSO and LIMMA analysis simultaneously, genes with best fold change or λ was defined as a valuable biomarker. The samples used in signature building and validation must have adopted ACT, with clear RFS time and the gene expression information we needed. Thus, the sample size of the constructing is 146. The detailed demographical information is listed in Supplementary Table 3.

### Statistics for Classification, Prediction and Validation in the GEO and TCGA Series

We built a risk score using the formula of *CD36*, *ITGA3*, *FLT3*, *CR2*, *IL7*, *CD2*, and *CD55* expression by the method of LASSO Cox regression. Then, Patients were divided into high-risk and low-risk groups according to this specific risk score formula. Time-dependent receiver operating characteristic (ROC) analysis was implemented to calculate the area under the curve (AUC) for 1-, 3-, and 5-year overall survival (OS) and relapse-free survival (RFS) in order to confirm the accuracy of predicted response by signature using the “survivalROC” R package (18). Using the Kaplan-Meier (K-M) survival curve analyses and log-rank test, this research evaluated the prognostic significance of this signature. Then, we plotted the distribution of patients’ risk score, survival and recurrence status to show the relationship between the risk score and patients’ response. A heatmap was constructed with cluster analysis in view of the gene expression difference, according to the risk score in the help of the “ComplexHeatmap” R package. To further investigate the classification reliability of the identified genes signature, this research verified it in GSE14333 and TCGA in the same protocol. The samples used in signature building and validation must have adopted ACT, with clear RFS time and the gene expression information we needed. Thus, the sample sizes were 63 and 150 for signature building and validation, respectively. The detailed demographical information is listed in Supplementary Table 3.

### Functional Enrichment Analysis

Functional enrichment analysis of KEGG pathway was performed to determine significantly enriched KEGG pathways of genes correlated with the signature using the ClueGO plugin (version 2.5.6) in Cytoscape limited in biological processes (19) and R software. The results of functional map and clusters of KEGG enrichment were obtained and visualized using a two-sided hypergeometric test with Bonferroni step down correction and kappa score threshold of 0.4, and limited in the level intervals 3–8 with *p* ≤ 0.05. Biological pathways with *p* < 0.05 was considered as significant using functional annotation chart options with the whole human genome as background.

### Correlation Between the Prognostic Signature and Other Clinicopathological Characteristics and Clinical Usefulness

The K-M survival analysis was performed on designated subtypes of different clinicopathological features, including gender, age, tumor site, pathological T stage, pathological N stage, MMR status, TP53 mutation status, KRAS mutation status and BRAF mutation status, to further testing the applicability of gene signatures. Univariable and multivariable cox regression analyses were adopted to calculate and validate the influence of variables, with *p* ≤ 0.05. This research found that pathological N stage was independent prognostic factors that could be used in combination with signature to predict RFS and OS after ACT. Based on the multivariable cox regression analysis results, two nomograms integrating clinicopathological parameters with signature were formulated by applying the “rms” R package. The overall points for each patient in the training and validation cohorts were calculated using founded nomograms. Decision curve analysis (DCA) incorporates a risk prediction model into clinical approach to evaluate a predictive model and visualizes the latent profit of therapy (20).

#### Statistical Analysis

All statistical analyses were performed with use of R (version 3.5.1, www.r-project.org). All statistical tests were two-sided, and *p* values < 0.05 were considered statistically significant.

## Results

### Concentration on the HCL Signaling Pathway

**FIGURE 2:**
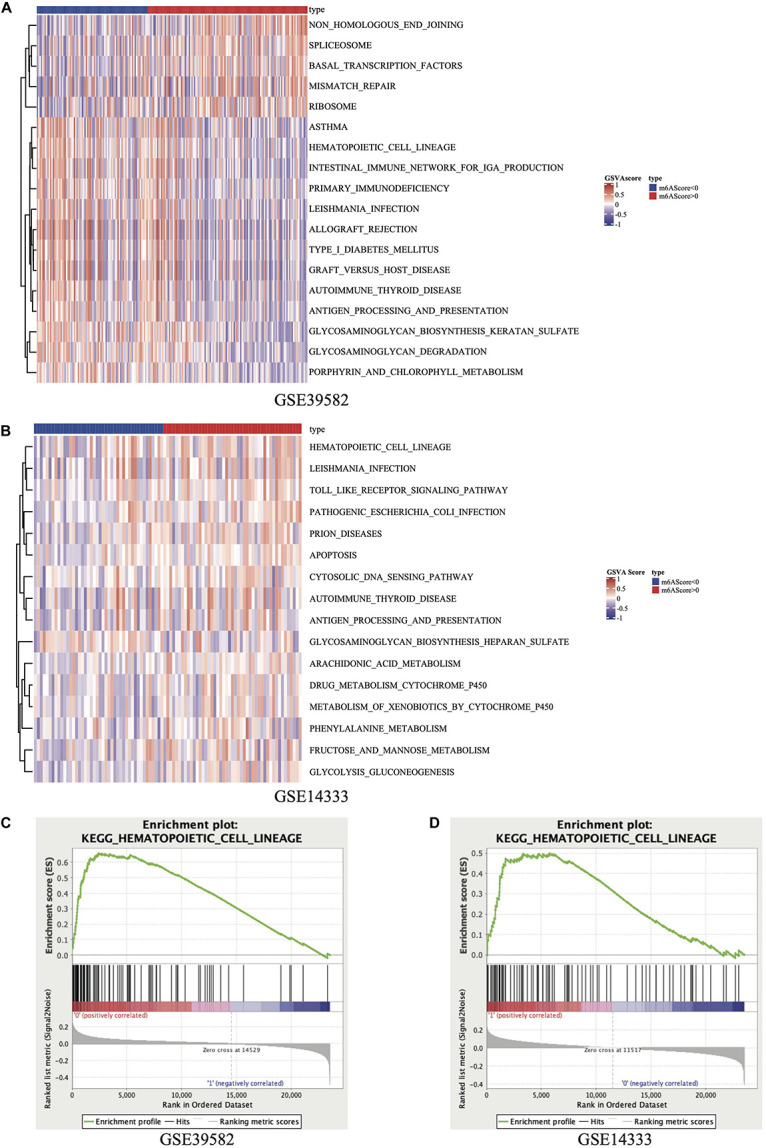
Heatmap of GSVA results in GSE39582 **(A)** and GSE14333 **(B)**. Results of GSEA on KEGG_HEMATOPOIETIC_CELL_LINEAGE pathway in GSE39582 **(C)** and GSE14333 **(D)**.

The R package of FactoMineR was used to calculate m^6^A score based on the expression of 21 m^6^A regulators and to classify patients with qualitatively different m^6^A modification patterns (Supplementary Figure 1). The demographical information and Three databases used in this research and the sample size are listed in the Supplementary Table 3. The m^6^A score of patients in the GSE39582 and GSE14333 was calculated and displayed in Supplementary Table 4. This study carried out GSVA of KEGG gene sets in 2 independent GEO data sets: GSE39582 and GSE14333. The results displayed in heatmap (Figures 2A,B) and Supplementary Table 5, concentrated on the active HCL signaling pathway and significantly focused on the m^6^A modification subtype groups. Meanwhile, we performed a GSEA of the KEGG gene sets and found that HCL was noticeably enriched in 2 data sets (Figures 2C,D). After fully considering the results of GSVA and GSEA, we selected the KEGG pathway with logFC >0.15, Enrichment Score >0.65 in GSE39582 and logFC >0.1, Enrichment Score >0.45 in GSE14333, respectively. Comprehensively, the results of the GSVA and GSEA showed that genes in HCL signaling pathway might be related to m^6^A modification levels in stage III CRC patients.

### Development of Efficacy Evaluation Signature From GSE39582 Set

**FIGURE 3:**
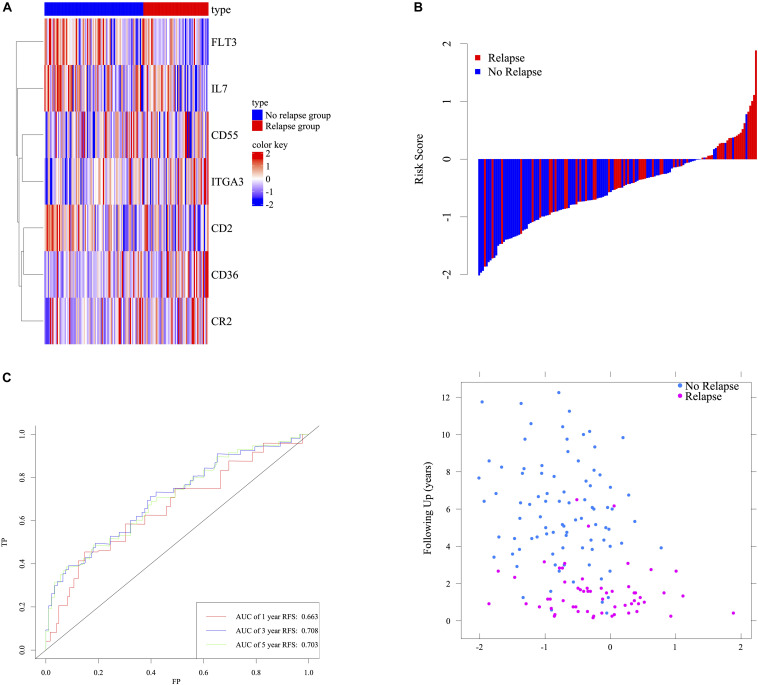
Determination and analysis of the seven-HCL-related signature in GSE39582 cohort. The expression pattern of the seven-HCL-related signature **(A)**. The distribution of patients’ risk score and relapse status **(B)**. Time dependent ROC curves at 1, 3, and 5 years **(C)**.

LASSO cox regression analyses were used to screen 87 response-related HCL genes in stage III CRC patients with ACT. The analysis of discrepantly expressed HCL-related genes (DEHG) between early relapse and long-term survival groups was performed using LIMMA method. 42 HCL-related genes were found associated with stage III CRC patients’ survival in LASSO analyses (Supplementary Figure 2). Besides, 4 genes (Supplementary Table 6) expressed differentially using LIMMA method and the heatmap of those genes was displayed in Supplementary Figure 3. Screening LASSO results by DEHG, it was found that 7 HCL-related genes were differentially expressed in patients with different ACT responses. The risk score formula of the gene marker predicting the ACT response was calculated by weighting the relative expression of each prognostic gene and its associated expression value through the LASSO cox regression coefficient of gene. Patients were divided into high-risk and low-risk groups according to this specific risk score formula. The formula was as follows: 0.64973 ^∗^ *CD36* expression + 0.50566 ^∗^ *ITGA3* expression – 1.06119 ^∗^ *FLT3* expression + 0.43809 ^∗^ *CR2* expression – 0.24174 ^∗^ *IL7* expression − 0.43412 ^∗^ *CD2* expression – 0.03472 ^∗^ *CD55* expression. According to the signature, stage III CRC patients were divided into low-risk and high-risk group using the value with acceptable sensitivity and specificity as the cutoff point. Based on the Youden’s index in the ROC curve, a simple cutoff point of this signature could be figured out, which is −1.193094. If the signature score exceeds the cutoff point, patents will be classified as the high risk and vice versa. The heatmap of the signature was displayed in Figure 3A. The distribution of relapse after ACT related to risk scores was shown in Figure 3B, suggesting that patients with lower risk scores tend to have better ACT response than others. Time-dependent ROC analysis at 1-, 3-, and 5-year RFS after resection were conducted to distinguish how accurate the signature was at predicting prognosis conditions. The AUC was 0.663, 0.708, and 0.708 at the survival time of 1, 3, and 5 years in GSE39582, respectively (Figure 3C). The results reflected our signature could predict the ACT effects among patients with stage III CRC.

### Validation of the Signature in GEO and TCGA Datasets

We validated the HCL-related signature based on the cases from GSE14333 and TCGA. In order to explore the effect of signature on relapse and survival outcomes, this research subsequently validated the results in GSE39582 OS cohorts. Risk scores were calculated according to the HCL-related signature.

**FIGURE 4:**
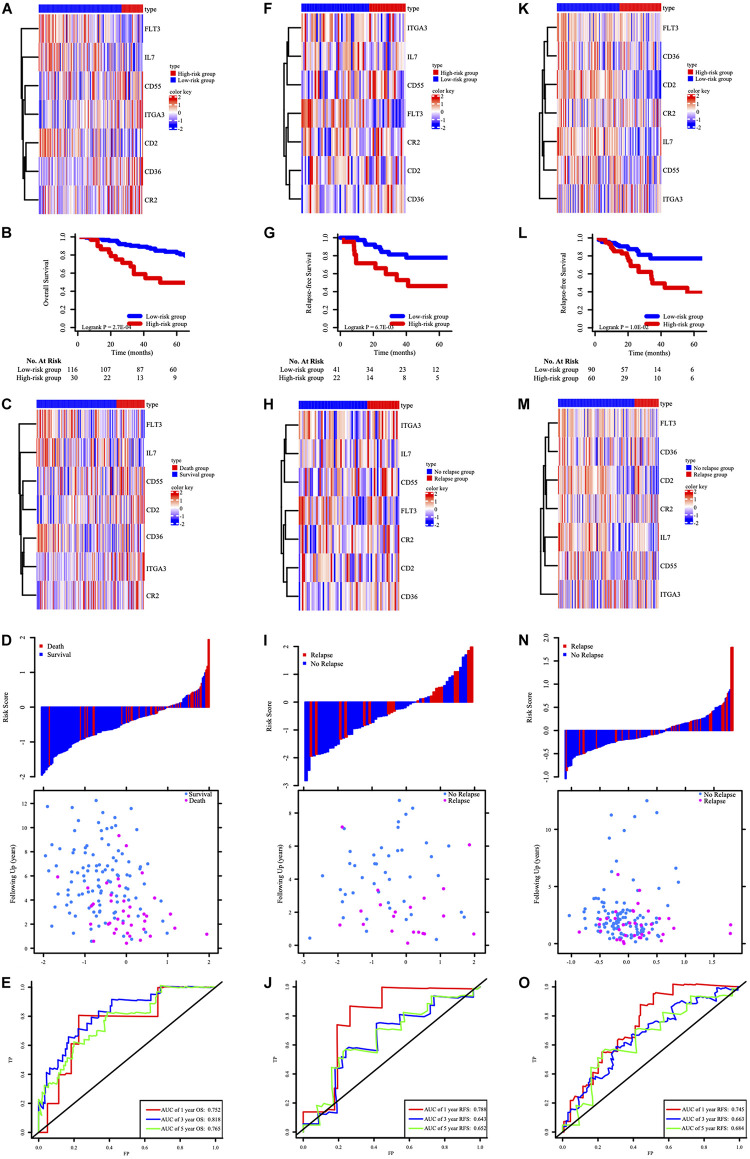
The expression pattern of the seven-HCL-related signature based on risk groups in the GSE39582 OS cohort **(A)**, GSE14333 RFS cohorts **(F)**, and TCGA RFS cohorts **(K)**. The K-M survival curves in the GSE39582 OS cohort **(B)**, GSE14333 RFS cohorts **(G)**, and TCGA RFS cohorts **(L)**. The expression pattern of the seven-HCL-related signature based on survival or relapse status in the GSE39582 OS cohort **(C)**, GSE14333 RFS cohorts **(H)**, and TCGA RFS cohorts **(M)**. The distribution of patients’ risk score and relapse status in GSE14333 **(I)**, and TCGA **(N)**. The distribution of patients’ risk score and survival status in GSE39582 **(D)**. AUC values of ROC for predicting response of ACT in stage III CRC patients in the GSE39582 OS cohort **(E)**, GSE14333 RFS cohorts **(J)**, and TCGA RFS cohorts **(O)**.

In GSE39582 OS cohorts, the clustered different expression patterns of the seven genes between low-, high-risk and survival, death group were analyzed and shown in Figures 4A,D. Compared to the patients in high risk group, the death rates after ACT for patients in the low-risk group were remarkably lower (Figure 4B). Distribution of the survival time and status among the 146 stage III CRC patients with ACT in GSE39582 was displayed in Figure 4C. Time-dependent ROC analyses at 1, 3, and 5 years were conducted to assess the predictive accuracy of the seven-HCL-based classifier and AUC of ROC proved that the classifier had excellent predictive preciseness (Figure 4E). In GSE14333 and TCGA cohorts, patients were also divided into low-risk and high-risk group in the same way as GSE39582. The K-M survival analysis (Figures 4G,L) and distribution of patients’ survival time and status (Figures 4I,N) showed that this classifier’s performance in predicting the RFS after ACT was consistent in external validation cohorts. The heatmap showed that the expression patterns of seven-HCL-related genes were the same regardless of whether they were grouped by risk or recurrence (Figures 4F,K,H,M). Besides, the AUC of the time-dependent ROC analysis proved that the classifier had good predictive specificity and sensitivity in 1-, 3-, and 5-year survival for GSE14333 and TCGA (Figures 4J,O).

### Distinguishing Ability of Signature on Chemotherapy Response and Potential Biological Function

**FIGURE 5:**
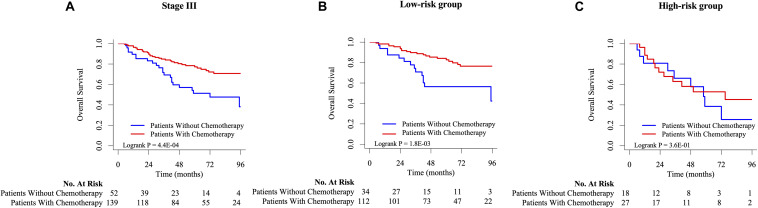
The K-M survival analysis between chemotherapy and non-chemotherapy group on stage III **(A)**, low-risk **(B)**, and high-risk **(C)** CRC patients in GSE39582.

As shown in Figure 5A, there was a significant difference in OS between patients with stage III CRC receiving chemotherapy and those without chemotherapy. After that, this study used signature to stratify the risk of stage III CRC patients and performed K-M survival analysis and log-rank test for chemotherapy factors in high-risk and low-risk groups, respectively (Figures 5B,C). ACT for low-risk group could help improve the stage III CRC patients’ OS, while ACT for high-risk groups might not have no significance for the survival. This result supported that stage III CRC patients with low-ACT-sensitivity were classified into high-risk groups and those with high-ACT-sensitivity were classified into low-risk groups. According to functional enrichment analysis of KEGG pathway (Supplementary Figures 4B–D), seven HCL-related biomarkers might play a role through PI3K-Akt signaling pathway and ECM-receptor interaction pathway. The protein-protein interaction network illustrated that these biomarkers also had strong and complex connections with each other (Supplementary Figure 4A).

### Stratification Analysis

**FIGURE 6:**
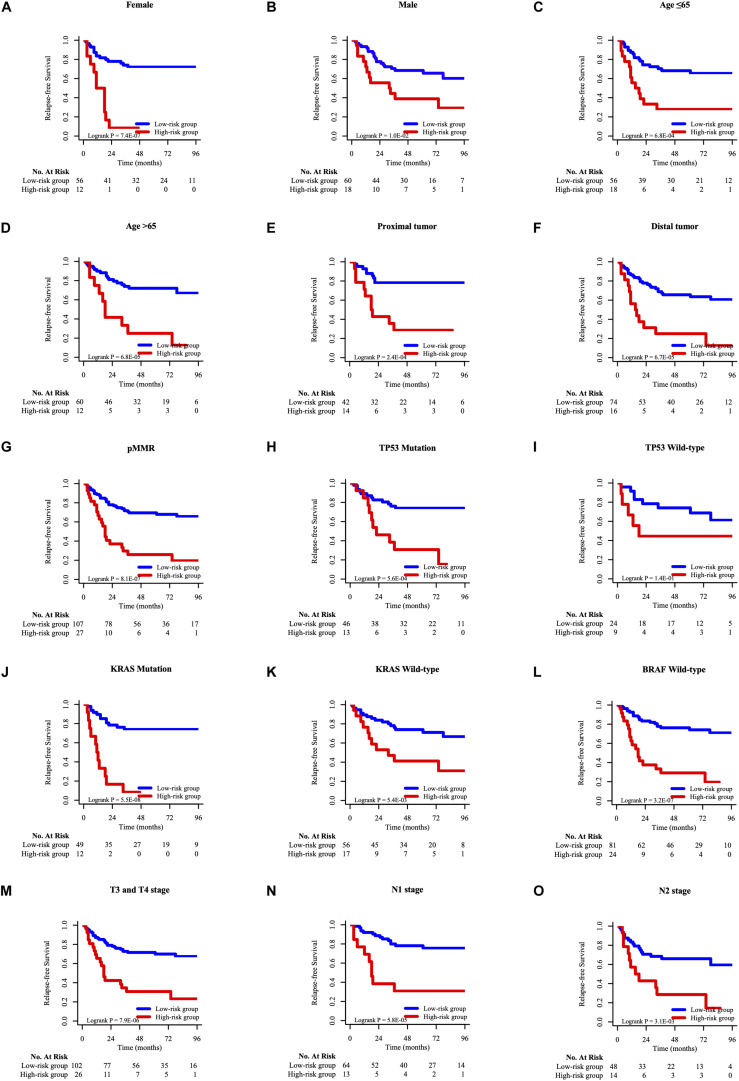
The K–M survival curves of overall survival between high-risk and low-risk group of different clinicopathological features, including gender **(A,B)**, age **(C,D)**, tumor site **(E,F)**, MMR status **(G)**, TP53 mutation status **(H,I)**, KRAS mutation status **(J,K),** BRAF mutation status **(L)**, pathological T stage **(M)**, pathological N stage **(N,O)**.

To determine whether the prognostic model can apply to other clinical factors, stratification analysis was performed according to age, sex, tumor site, pathological T stage, pathological N stage, MMR status, TP53 mutation status, KRAS mutation status, and BRAF mutation status. As the result of K-M analysis, seven-HCL-related-gene signature was quite meaningful in most clinically subgroups, although it did not reach the statistical difference in some factors due to the limitation of the number of cases (Figure 6 and Supplementary Figure 6).

### Setting Up a Clinical Prediction Model

**FIGURE 7:**
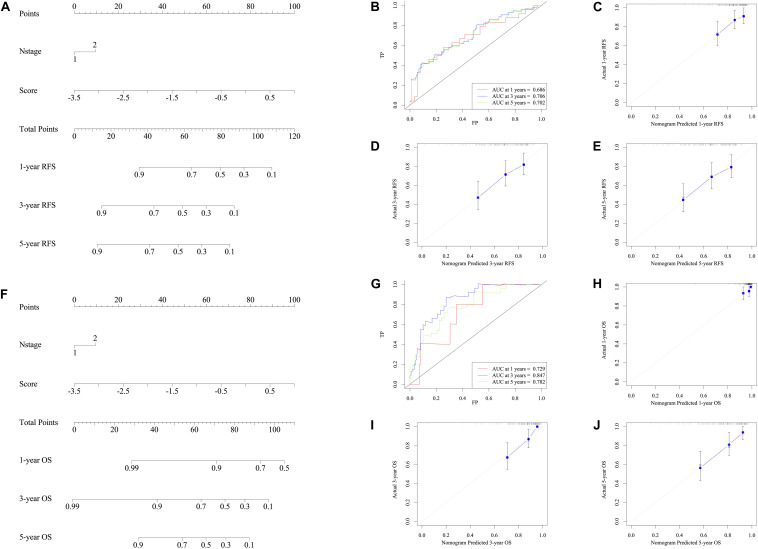
Nomograms convey the results of prognostic models using the seven-HCL-related signature and pathology N stage to predict RFS of patients with CRC in TCGA cohort. The AUC at 1-year prediction was 0.686 **(A,B)**. The *x*-axis is nomogram-predicted probability of survival and *y*-axis is actual survival. The reference line is 45∘ and indicates perfect calibration (**C** as 1-year, **D** as 3-year, and **E** as 5-year). Nomograms convey the results of prognostic models using the seven-HCL-related signature and pathology N stage to predict OS of patients with CRC in TCGA cohort. The AUC at 1-year prediction was 0.729 **(F,G)**. The *x*-axis is nomogram-predicted probability of survival and *y*-axis is actual survival. The reference line is 45∘ and indicates perfect calibration (**H** as 1-year, **I** as 3-year, and **J** as 5-year).

**FIGURE 8:**
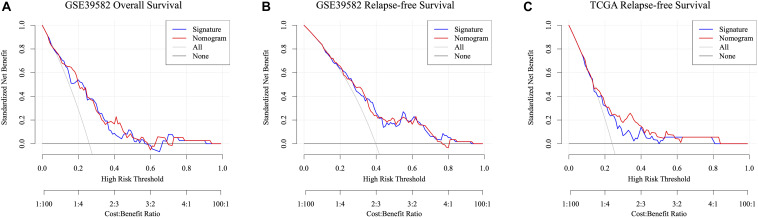
Decision curve analysis of the nomogram and signature for predicting ACT-response of stage III CRC patients in the GSE39582 RFS cohort **(A)**, GSE39582 OS cohorts **(B)**, and TCGA RFS cohorts **(C)**. The gray line and black line represent the assumption regarding all patients with and without risk stratification, respectively. The red line represents the nomogram, and the blue line represents the signature.

Taking the univariable and multivariable cox regression model in GSE39582 cohort (Supplementary Tables 7, 8), this study constructed two nomograms to satisfy the needs of clinicians to quantify the prognosis of stage III CRC patients (Figure 7A referred to RFS and Figure 7F referred to OS). To ensure its efficacy in predicting RFS and OS, time-dependent ROC was applied, which suggested that the nomogram had good prognostic accuracy (Figures 7B,G). The sensitivity of nomogram in predicating the relapse status in the GSE39582 is 0.5787923. Calibration curves of the nomogram revealed no deviations from the reference line (Figures 7C,H as 1-year, Figures 7D,I as 3-year, Figures 7E,J as 5-year). To verify this conclusion, the same protocol was duplicated in the TCGA RFS cohort, shown in Supplementary Figure 6 and Supplementary Table 9. The sensitivity of nomogram in predicting the relapse status in TCGA cohort is 0.6179945. The DCA curves for the developed nomogram and signature in GSE39582 and TCGA cohorts were shown in Figure 8. Both DCA showed high net benefits, so it had excellent clinical outcome values, DCA of nomograms described that integration of clinical and gene expression pattern was more reliable than gene signature. Detailed standardized net benefits were listed in Supplementary Table 10.

## Discussion

Radical surgery combined with ACT is the prominent treatment to enhance the survival of patients with stage III CRC. However, for stage III CRC patients, there is a lack of molecular markers for predicting chemotherapy response and clinical prognosis. In this research, we put forward the idea that m6A modifications might be determinant in the ACT response of patients with stage III CRC (21). Then, a novel prognostic predictive signature in view of 7 HCL-related genes was formulated based on GSE39582 and GSE14333 cohorts. The divergence of ACT effects between low-risk and high-risk patient with stage III CRC was fully displayed in several methods. The prognostic signature has also been validated in GSE14333 and TCGA cohort. Furthermore, combing the pathological N stage and the signature, two nomograms have been set up to help clinician predict ACT response of stage III CRC patients.

The genes in the signature are closely related to the cancer and might be the potential treatment targets. Proteins in HCL pathway, like *CR2*, have already become biomarkers in the treatment and classification of hematologic tumor (22, 23). *CD2* was also reported to be highly associated with acute promyelocytic leukemia (APL). The expression of *CD2* was also considered as a prognostic factor in the APL (24). According to the pervious researches, the expression level of *CD2* and *CR2* demonstrated the status of our immune system and other immunological diseases. Our work indicated that *CD2* and *CR2* might account for the resistance and ineffectiveness of ACT. The relationship between those two genes and ACT effects need to be further confirmed in the molecular level. Recently, (25) also demonstrated those proteins like *IL7* may affect the formation and metastasis of breast cancer. Based on the *IL7* pathway, signaling cytokine receptor was established to improve the effects of the CAR-T therapy in preclinical tumor models (26). Our work also revealed the potential of improving ACT sensitivity via *IL7* pathway. Talking about *ITGA3*, this gene priorly was priorly found to predict the relapse of right-side colon cancer in stage II (27). Statistics disclosed the relationship between the *ITGA3* integrin and disease-free survival in patients with colorectal tumors (28). Researchers also established a link between miR-124 and anoikis susceptibility and proved that a miR-124/*ITGA3* axis could be a potential target for the treatment of metastatic CRC (29). As an important gene in AML, *FLT3* mutation happened in almost 30% AML cases and the mutation of *FLT3* kept changing in the processing of AML and also showed poor prognosis in AML patient (30). Patients with metastatic CRC and *FLT3* translocation might be sensitive to sorafenib treatment (31). Combing the pervious study and our outcome, the value of the *FLT3* in predicting the relapse and survival of tumor patients have been explored. Experiments for the molecular pathway of the *FLT3* and tumor progress should be set off. Researches into the expression level of *CD55* in CRC patients proved that patients with tumors expressing high levels of *CD55* had a significantly worse survival than patients with low *CD55* levels (32), which means the expression of *CD55* may serve as a marker for the CRC patients. *CD36* is a cell adhesion receptor and it was reported that it could modulate the vascularization of tumor tissues. *CD36* expression might decrease stromal vascularization which contributed to better prognosis of colon cancer (33). *CD36* is the upstream regulator of the PPAR signaling pathway, which can inhibit the procession of CRC. Generally speaking, the genes, figured out in this research, have been proved by others to be associated with the progress and prognosis of hematologic tumors and other solid tumors. There are few articles in CRC treatment and ACT sensitivity concerning genes like *CR2*, *CD2*, and *IL7*. Apart from the scientific values, genes like *IL7* can easily been tested in the current examine methods. Using our signature and biomarker doesn’t need to develop new testing method and antibody. Low cost and high sensitivity are one of the advantages of our research.

There are indeed some limitations in this study. On the one hand, our study was based on the data from public datasets without testing *in vitro* and *in vivo*. Further study is needed to validate whether expression of HCL genes was associated with m6A methylation. On the other hand, the sample capacity of our research is relatively small. Besides, our external confirmation cohorts are mainly from the same race. Thus, more patients’ data are needed for our further research and confirmation.

## Conclusion

In conclusion, we developed a seven-HCL-related mRNA signature composed of various regulation mRNA that effectively classify CRC patients into low-risk group (with high ACT sensitivity) and high-risk groups (with low ACT sensitivity). Application of the signature in clinical treatments should also be further observed to verify the validity of our findings.

## Data Availability Statement

The datasets presented in this study can be found in online repositories. The names of the repository/repositories and accession number(s) can be found in the article/ Supplementary Material.

## Author Contributions

ZZ, SM, and RG had the idea for this study. XZ and LH supervised the acquisition of the data. SM, ZZ, and WD undertook the statistical analysis. RW and LZ provided statistical advice. All authors contributed to interpretation of the results, approved the final version of the manuscript, and including the authorship list. SM, ZZ, and RG wrote the manuscript. LZ, RW, and GC revised the manuscript and other authors contributed to the content.

## Conflict of Interest

The authors declare that the research was conducted in the absence of any commercial or financial relationships that could be construed as a potential conflict of interest.

## Footnotes

1 http://www.ncbi.nlm.nih.gov/geo/

2 http://software.broadinstitute.org/gsea/index.jsp

3 https://tcga.xenahubs.net/

## Supplementary Material

Supplementary Figure 1 Results of PCA in view of patients from GSE39582 and 21 m^6^A regulators **(A,B)**. Consensus index after regrouping cases by PCA **(C)**. Heatmap of 21 m^6^A regulators expression in GSE39582 **(D)**.

Supplementary Figure 2 Results of LASSO lambda filtering **(A)**. Relationship between LASSO regression coefficient and lambda **(B)**.

Supplementary Figure 3 The heatmap of four genes found differentially expressed using LIMMA method.

Supplementary Figure 4 The protein-protein interaction network **(A)**. Dot plot of the functional enrichment analysis of KEGG pathway **(B)**. Protein and pathway relationship network **(C)**. Pathway and pathway interaction network **(D)**.

Supplementary Figure 5 The K–M survival curves of overall survival between high-risk and low-risk group of different clinicopathological features, including gender **(A,B)**, age **(C,D)**, tumor site **(E,F)**, MMR status **(G)**, TP53 mutation status **(H,I)**, KRAS mutation status **(J,K)**, BRAF mutation status **(L)**, pathological T stage **(M)**, pathological N stage **(N,O)**.

Supplementary Figure 6 Nomograms convey the results of prognostic models using the seven-HCL-related signature and pathology N stage to predict RFS of patients with CRC in TCGA cohort. The AUC at 1-year prediction was 0.759 **(A,B)**. The *x*-axis is nomogram-predicted probability of survival and *y*-axis is actual survival. The reference line is 45∘ and indicates perfect calibration (**C** as 1-year, **D** as 3-year, **E** as 5-year).

Supplementary Table 1 21 genes of m^6^A regulators list obtained from the GSEA database.

Supplementary Table 2 87 HCL genes list obtained from the GSEA database.

Supplementary Table 3 Demographic information of patients with stage III CRC in GSE39582, GSE14333, and TCGA cohorts.

Supplementary Table 4 Result scores of PCA in GSE39582 and GSE14333.

Supplementary Table 5 Result scores of GSVA in GSE39582 and GSE14333.

Supplementary Table 6 Gene list through LASSO analyses and LIMMA method.

Supplementary Table 7 Univariable and multivariable Cox regression model analyses of relapse-free survival in GSE39582 cohort.

Supplementary Table 8 Univariable and multivariable Cox regression model analyses of overall survival in GSE39582 cohort.

Supplementary Table 9 Univariable and multivariable Cox regression model analyses of relapse-free survival in TCGA cohort.

Supplementary Table 10 Standardized net benefit for specific optimal thresholds using DCA based on GSE39582 and TCGA.

## Abbreviations

CRC: colorectal cancer
ACT: adjuvant chemotherapy
PNI: perineural invasion
m^6^A: N6-methyladenosinel
SNPs: single-nucleotide polymorphisms
HSCs: Hematopoietic stem cells
HCL: Hematopoietic cell lineage
PCA: principal component analysis
GEO: Gene Expression Omnibus
TCGA: the Cancer Genome Atlas
DCA: decision curve analysis
GSVA: Gene Set Variation Analysis
GSEA: Gene Set Enrichment Analysis
KEGG: Kyoto encyclopedia of genes and genomes
AUC: the area under the curve
ROC: receiver operating characteristic
LIMMA: linear models for microarray data
DEGs: differentially expressed genes
LASSO: least absolute shrink-age and selection operator
RFS: relapse-free survival
OS: overall survival
K-M: Kaplan–Meier
DEHG: discrepantly expressed HCL-related genes.
