# Supplementary material for: Hematopoietic Gene Expression Regulation Through m6A Methylation Predicts Prognosis in Stage III Colorectal Cancer
Source: Front Oncol. 2020 Sep 30;10:572708. doi: 10.3389/fonc.2020.572708 (PMC7556240; doi:10.3389/fonc.2020.572708)
Supplement: Supplementary Figure 1 — Results of PCA in view of patients from GSE39582 and 21 m6A regulators (A,B). Consensus index after regrouping cases by PCA (C). Heatmap of 21 m6A regulators expression in GSE39582 (D). [file Data_Sheet_1.zip › Data Sheet 1/Supplementary Materials/Figure S5.pdf]

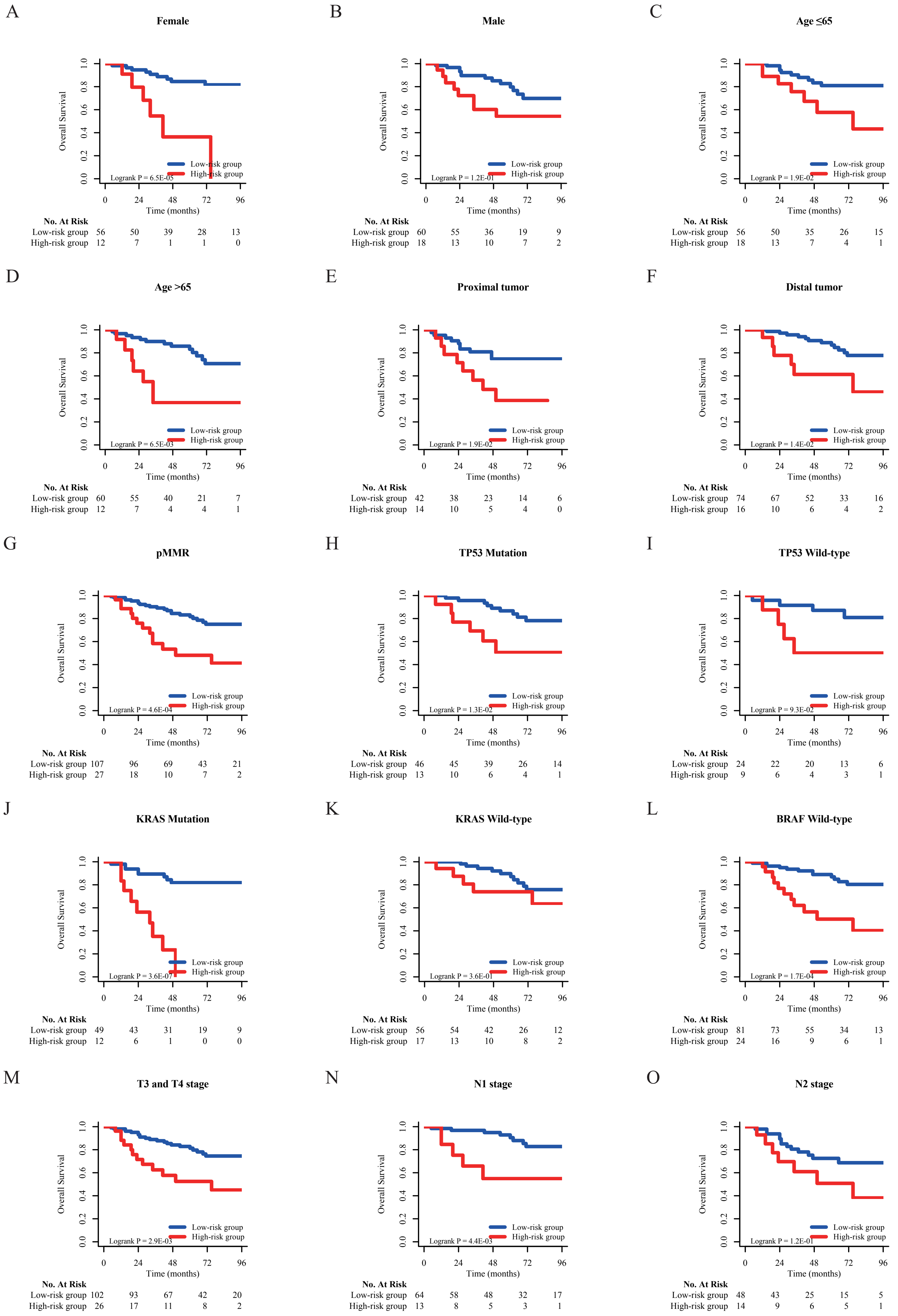
A
Female
Overall Survival
Time (months)
Low-risk group
High-risk group
Logrank P = 6.5E-05
No. At Risk
Low-risk group 56 50 39 28 13
High-risk group 12 7 1 1 0
B
Male
Overall Survival
Time (months)
Low-risk group
High-risk group
Logrank P = 1.2E-01
No. At Risk
Low-risk group 60 55 36 19 9
High-risk group 18 13 10 7 2
C
Age ≤65
Overall Survival
Time (months)
Low-risk group
High-risk group
Logrank P = 1.9E-02
No. At Risk
Low-risk group 56 50 35 26 15
High-risk group 18 13 7 4 1
D
Age >65
Overall Survival
Time (months)
Low-risk group
High-risk group
Logrank P = 6.5E-03
No. At Risk
Low-risk group 60 55 40 21 7
High-risk group 12 7 4 4 1
E
Proximal tumor
Overall Survival
Time (months)
Low-risk group
High-risk group
Logrank P = 1.9E-02
No. At Risk
Low-risk group 42 38 23 14 6
High-risk group 14 10 5 4 0
F
Distal tumor
Overall Survival
Time (months)
Low-risk group
High-risk group
Logrank P = 1.4E-02
No. At Risk
Low-risk group 74 67 52 33 16
High-risk group 16 10 6 4 2
G
pMMR
Overall Survival
Time (months)
Low-risk group
High-risk group
Logrank P = 4.6E-04
No. At Risk
Low-risk group 107 96 69 43 21
High-risk group 27 18 10 7 2
H
TP53 Mutation
Overall Survival
Time (months)
Low-risk group
High-risk group
Logrank P = 1.3E-02
No. At Risk
Low-risk group 46 45 39 26 14
High-risk group 13 10 6 4 1
I
TP53 Wild-type
Overall Survival
Time (months)
Low-risk group
High-risk group
Logrank P = 9.3E-02
No. At Risk
Low-risk group 24 22 20 13 6
High-risk group 9 6 4 3 1
J
KRAS Mutation
Overall Survival
Time (months)
Low-risk group
High-risk group
Logrank P = 3.6E-07
No. At Risk
Low-risk group 49 43 31 19 9
High-risk group 12 6 1 0 0
K
KRAS Wild-type
Overall Survival
Time (months)
Low-risk group
High-risk group
Logrank P = 3.6E-01
No. At Risk
Low-risk group 56 54 42 26 12
High-risk group 17 13 10 8 2
L
BRAF Wild-type
Overall Survival
Time (months)
Low-risk group
High-risk group
Logrank P = 1.7E-04
No. At Risk
Low-risk group 81 73 55 34 13
High-risk group 24 16 9 6 1
M
T3 and T4 stage
Overall Survival
Time (months)
Low-risk group
High-risk group
Logrank P = 2.9E-03
No. At Risk
Low-risk group 102 93 67 42 20
High-risk group 26 17 11 8 2
N
N1 stage
Overall Survival
Time (months)
Low-risk group
High-risk group
Logrank P = 4.4E-03
No. At Risk
Low-risk group 64 58 48 32 17
High-risk group 13 8 5 3 1
O
N2 stage
Overall Survival
Time (months)
Low-risk group
High-risk group
Logrank P = 1.2E-01
No. At Risk
Low-risk group 48 43 25 15 5
High-risk group 14 9 6 5 1
