# Supplementary material for: Hematopoietic Gene Expression Regulation Through m6A Methylation Predicts Prognosis in Stage III Colorectal Cancer
Source: Front Oncol. 2020 Sep 30;10:572708. doi: 10.3389/fonc.2020.572708 (PMC7556240; doi:10.3389/fonc.2020.572708)
Supplement: Supplementary Figure 1 — Results of PCA in view of patients from GSE39582 and 21 m6A regulators (A,B). Consensus index after regrouping cases by PCA (C). Heatmap of 21 m6A regulators expression in GSE39582 (D). [file Data_Sheet_1.zip › Data Sheet 1/Supplementary Materials/Table S10.docx]

Supplementary Table 10

Standardized net benefit using the overall survival nomogram based on GSE39582 for specific optimal thresholds.

| Threshold | Standardized net benefit all | Standardized net benefit signature | Standardized net benefit nomogram |
| --- | --- | --- | --- |
| 0.1 | 0.684 | 0.731 | 0.719 |
| 0.2 | 0.289 | 0.539 | 0.507 |
| 0.3 | 0.35 | 0.335 | 0.278 |
| 0.4 |  | 0.088 | 0.167 |
| 0.5 |  | 0.079 | 0.053 |
| 0.6 |  | -0.013 | -0.053 |
| 0.7 |  | 0.079 | -0.009 |
| 0.8 |  | 0.026 | 0.026 |
| 0.9 |  | 0.026 | 0.026 |
| 0.99 |  | 0 | 0 |

Standardized net benefit using the relapse-free survival nomogram based on GSE39582 for specific optimal thresholds.

| Threshold | Standardized net benefit all | Standardized net benefit signature | Standardized net benefit nomogram |
| --- | --- | --- | --- |
| 0.1 | 0.831 | 0.837 | 0.837 |
| 0.2 | 0.621 | 0.634 | 0.655 |
| 0.3 | 0.35 | 0.433 | 0.478 |
| 0.4 |  | 0.259 | 0.241 |
| 0.5 |  | 0.172 | 0.207 |
| 0.6 |  | 0.207 | 0.164 |
| 0.7 |  | 0.063 | 0.098 |
| 0.8 |  | 0.052 | -0.034 |
| 0.9 |  | 0.017 | 0.017 |
| 0.99 |  | 0 | 0 |

Standardized net benefit using the relapse-free survival nomogram based on TCGA for specific optimal thresholds.

| Threshold | Standardized net benefit all | Standardized net benefit signature | Standardized net benefit nomogram |
| --- | --- | --- | --- |
| 0.1 | 0.648 | 0.636 | 0.627 |
| 0.2 | 0.208 | 0.319 | 0.333 |
| 0.3 |  | 0.067 | 0.179 |
| 0.4 |  | 0.139 | 0.148 |
| 0.5 |  | 0 | 0.056 |
| 0.6 |  | 0.056 | 0.042 |
| 0.7 |  | 0.056 | 0.056 |
| 0.8 |  | 0.028 | 0.056 |
| 0.9 |  | 0 | 0 |
