# Supplementary material for: Hematopoietic Gene Expression Regulation Through m6A Methylation Predicts Prognosis in Stage III Colorectal Cancer
Source: Front Oncol. 2020 Sep 30;10:572708. doi: 10.3389/fonc.2020.572708 (PMC7556240; doi:10.3389/fonc.2020.572708)
Supplement: Supplementary Figure 1 — Results of PCA in view of patients from GSE39582 and 21 m6A regulators (A,B). Consensus index after regrouping cases by PCA (C). Heatmap of 21 m6A regulators expression in GSE39582 (D). [file Data_Sheet_1.zip › Data Sheet 1/Supplementary Materials/Table S9.docx]

| Supplementary Table 9 Univariable and multivariable Cox regression model analyses of relapse-free survival in TCGA cohort | | | | | | | |
| --- | --- | --- | --- | --- | --- | --- | --- |
| Variables | Univariable analysis | | |  | Multivariable analysis | | |
|  | HR | 95% CI | P-value |  | HR | 95% CI | P-value |
| Gender |  |  | 0.07 |  |  |  | <0.001 |
| Female | 1 |  |  |  | 1 |  |  |
| Male | 1.859 | 0.950-3.639 |  |  | 3.001 | 1.337-6.733 |  |
| Age |  |  | 0.09 |  |  |  | 0.15 |
| >65 | 1 |  |  |  | 1 |  |  |
| ≤65 | 1.789 | 0.905-3.535 |  |  | 1.827 | 0.799-4.177 |  |
| Tumor site |  |  | 0.74 |  |  |  | 0.42 |
| Colon | 1 |  |  |  | 1 |  |  |
| Rectum | 1.134 | 0.546-2.354 |  |  | 1.400 | 0.612-3.201 |  |
| Venous invasion |  |  | 0.11 |  |  |  | 0.10 |
| No | 1 |  |  |  | 1 |  |  |
| Yes | 1.870 | 0.883-3.960 |  |  | 2.185 | 0.865-5.521 |  |
| Histological type |  |  | 0.25 |  |  |  | 0.74 |
| Adenocarcinoma | 1 |  |  |  | 1 |  |  |
| Mucinous adenocarcinoma | 1.725 | 0.713- 4.176 |  |  | 1.239 | 0.353-4.346 |  |
| Lymphatic invasion |  |  | 0.31 |  |  |  | 0.47 |
| No | 1 |  |  |  | 1 |  |  |
| Yes | 1.464 | 0.694-3.088 |  |  | 0.710 | 0.279-1.807 |  |
| T stage |  |  | 0.55 |  |  |  | 0.83 |
| T1 and T2 | 1 |  |  |  |  |  |  |
| T3 and T4 | 1.502 | 0.359-6.289 |  |  | 1.251 | 0.164-9.568 |  |
| N stage |  |  | <0.001 |  |  |  | <0.001 |
| N1 | 1 |  |  |  | 1 |  |  |
| N2 | 1.721 | 1.342-5.008 |  |  | 2.905 | 1.294-6.524 |  |
| HR, Hazard ratio; CI, Confidence interval | | | | | | | |
